# Elevated Adipsin and Reduced C5a Levels in the Maternal Serum and Follicular Fluid During Implantation Are Associated With Successful Pregnancy in Obese Women

**DOI:** 10.3389/fendo.2022.918320

**Published:** 2022-07-13

**Authors:** Manjunath Ramanjaneya, Ilhame Diboun, Najeha Rizwana, Yaser Dajani, Lina Ahmed, Alexandra E. Butler, Thoraya Ali Almarzooqi, Mohammed Shahata, Moza Khalaf Al Bader, Einas Elgassim, Hasan Burjaq, Stephen L. Atkin, Abdul-Badi Abou-Samra, Mohamed A. Elrayess

**Affiliations:** ^1^ Qatar Metabolic Institute, Hamad Medical Corporation, Doha, Qatar; ^2^ Translational Research Institute, Hamad Medical Corporation, Doha, Qatar; ^3^ College of Health and Life sciences, Doha, Qatar; ^4^ Biomedical Research Center (BRC), Qatar University, Doha, Qatar; ^5^ Reproductive Medicine, Doha, Qatar; ^6^ Department of Medicine, Doha, Qatar; ^7^ Post Graduate Studies and Research, Adliya, Bahrain; ^8^ Obstetrics and Gynecology Department, Women’s Hospital, Hamad Medical Corporation, Doha, Qatar; ^9^ QU Health, Qatar University, Doha, Qatar

**Keywords:** adipsin, follicular fluid (FF), *in vitro* fertilization (IVF), complement factors, embryos

## Abstract

**Introduction:**

Complement factors mediate the recruitment and activation of immune cells and are associated with metabolic changes during pregnancy. The aim of this study was to determine whether complement factors in the maternal serum and follicular fluid (FF) are associated with *in vitro* fertilization (IVF) outcomes in overweight/obese women.

**Methods:**

Forty overweight/obese (BMI = 30.8 ± 5.2 kg/m^2^) female patients, 33.6 ± 6.3 years old, undergoing IVF treatment for unexplained infertility were recruited. Baseline demographic information, including biochemical hormonal, metabolic, and inflammatory markers, and pregnancy outcome, was collected. Levels of 14 complement markers (C2, C4b, C5, C5a, C9, adipsin, mannose-binding lectin, C1q, C3, C3b/iC3b, C4, factor B, factor H, and properdin) were assessed in the serum and FF and compared to IVF outcome, inflammatory, and metabolic markers using multivariate and univariate models.

**Results:**

Out of 40 IVF cycles, 14 (35%) resulted in pregnancy. Compared to women with failed pregnancies, women with successful pregnancies had higher levels of adipsin in the serum and FF (*p* = 0.01) but lower C5a levels (*p* = 0.05). Serum adipsin levels were positively correlated with circulating levels of vitamin D (*R* = 0.5, *p* = 0.02), glucagon (*R* = 0.4, *p* = 0.03), leptin (*R* = 0.4, *p* = 0.01), resistin (*R* = 0.4, *p* = 0.02), and visfatin (*R* = 0.4, *p* = 0.02), but negatively correlated with total protein (*R* = −0.5, *p* = 0.03). Higher numbers of top-quality embryos were associated with increased levels of C3, properdin, C1q, factors H and B, C4, and adipsin, but with reduced C2 and C5a levels (*p* ≤ 0.01).

**Conclusions:**

Higher adipsin and lower C5a levels in the maternal serum during implantation are potential markers of successful outcome in obese women undergoing IVF-assisted pregnancies.

## Introduction

The global obesity epidemic has led to detrimental consequences on human bodily functions including reproductive health (1), with obesity being recognized as one of the leading causes for the decrease in fertility rate. Infertility affects between 8% and 12% of the reproductive age group population, and in certain regions, such as the Middle East and North Africa, a region that includes Qatar, the infertility rate is estimated to be as high as 30%. During the last decade, Qatar has seen a steady rise in the overweight/obese population and a decline in fertility rates among women (2). Obese women have a higher incidence of menstrual dysfunction, anovulation, pregnancy complications, polycystic ovary syndrome, and fertility issues. Furthermore, the rate of miscarriages increases as BMI increases (3). Excess adipose tissue in obese women secretes elevated inflammatory adipocytokines that alter the hormonal balance and cause reproductive disturbances. Elevated levels of inflammatory cytokines, together with elevated insulin secretion, induce hyperandrogenemia and promote granulosa cell apoptosis, which may affect both ovaries (4) and endometrium (5), causing fertility problems.

Complement proteins are secreted by the immune cells, which contribute to danger sensing by activating complement receptors on their target cells. Such activation participates in the type and magnitude of the immune response together with signaling pathways activated in response to pattern recognition receptors (6).

Adipsin, also known as complement factor D, is a member of the trypsin family of peptidases and is secreted mainly from adipocytes, monocytes, and macrophages (7, 8). It plays a critical role in the development of the C5–C9 membrane attack complex and the production of several signaling molecules, including the anaphylatoxins, C3a and C5a (9–11). It is also involved in the first step of activation of the alternate complement pathway (**Supplementary Figure 1**), where it produces the C3bBb complex (C3 convertase) by factor B and C3b. C3 convertase is responsible for cleaving C3a from C3 and releasing C3b (12). Adipsin regulates adipose tissue homeostasis and elevates glucose secretion (9). It also regulates the differentiation of adipocytes and promotes the accumulation of lipids, which is hypothesized to be a potential cause for the association of adipsin with metabolic disorders (13). Indeed, elevated adipsin levels have been associated with ischemia perfusion (14) and sepsis (15). Furthermore, increased levels of circulating adipsin were closely associated with polycystic ovary syndrome (16), mild cognitive impairment in type 2 diabetic mellitus patients (18), and coronary artery disease (17), suggesting adipsin as a promising biomarker for the diseases. C5a, on the other hand, is a very potent complement factor that plays a chemoattractant role by inducing the migration of many cells involved in the immune response and wound healing, including neutrophils and macrophages (19). It also links innate and adaptive immunity, extending its role in inflammation (19). Previous studies have suggested a role of C5a in the risk of diabetic kidney disease (20) and cardiovascular disease (21).

The plasma levels of adipsin and C5a were shown to be significantly elevated prior to delivery in pregnant women with preeclampsia (22); however, in healthy pregnant women, plasma adipsin and C5a were increased from the third trimester (23). As noted above, the main function of adipsin is to catalyze the breakdown of complement factor C3; therefore, adipsin may affect the downstream molecules such as C3a and C5a that have been shown to be increased in pregnancy, indicating that the complement system is activated during normal pregnancy (Karina 24).

The complement system exhibits both damaging and protective roles at the placental level. Activation of complement factors at the fetal–maternal interface protects against infectious agents and removes apoptotic and necrotic cells (25). However, various reports have implicated complement activation in the pathogenesis of adverse pregnancy outcomes (26–30). Despite evidence suggesting involvement of complement factors in obstetrics diseases, no study has investigated their concentrations with regard to *in vitro* fertilization (IVF) outcome. The hypothesis of this study was that complement factors are dysregulated in obese/overweight women who fail to have successful pregnancy following IVF treatment. To address this hypothesis, levels of 14 complement factors were assessed in the maternal serum and follicular fluid (FF) and compared in relation to IVF pregnancy outcome in overweight/obese women and other inflammatory markers.

## Materials and Methods

### Study Design

This was a prospective exploratory pilot cohort study and was performed from January 2017 to January 2018. Forty young overweight/obese Qatari female patients undergoing IVF for unexplained infertility were recruited at Hamad Medical Corporation. Inclusion criteria for the study are as follows: no concurrent illness, not on any medication for the preceding 9 months except fertility medications, and patient gave written informed consent. Exclusion criteria are as follows: women with diabetes, non-classical 21-hydroxylase deficiency, hyperprolactinemia, and Cushing’s disease, and women who had androgen-secreting tumors were excluded from the study. Demographics, anthropometrics, and medical history data were collected, including age, ethnicity, socioeconomic background, vital signs, height, weight, menstrual cycle, period of infertility, medications, complications, comorbidities, and family medical history. Study participants had no medical condition or illness and all women were on folic acid 400 mcg daily, but no other medication. Demographic data are shown in **Table 1**. Blood samples were collected at the beginning of the IVF cycle (taken in the follicular phase of the cycle) and just prior to hormonal downregulation, and immediately processed and stored at −80°C pending analysis.

**Table 1:** General characteristics of participants with successful or unsuccessful pregnancies.

Test	Variables	Total	Successful pregnancy *N* = 14	Unsuccessful pregnancy *N* = 26	*p*-value
Vital signs	Age (years)	34 (6.1)	31 (4.1)	35.5 (6.4)	0.011
	BMI (kg/m^2^)	33 (18.2)	31.15 (5.2)	34.60 (21.9)	0.936
	SBP (mmHg)	118.2 (12.5)	118 (12.1)	118.2 (13)	0.980
	DBP (mmHg)	74.8 (8.2)	73 (9.5)	75.72 (7.51)	0.382
Lipid profile	Cholesterol total (mmo/L)	4.81 (0.86)	4.18 (0.59)	4.91 (0.87)	0.234
	HDL-cholesterol (mmo/L)	1.38 (0.26)	1.21 (0.13)	1.40 (0.27)	0.444
	LDL-cholesterol (mmo/L)	2.97 (0.68)	2.57 (0.60)	3.03 (0.70)	0.476
	Triglyceride (mmo/L)	1.02 (0.36)	0.78 (0.16)	1.05 (0.37)	0.444
Blood sugar	Fasting blood glucose (nmol/L)	4.98 (0.45)	4.84 (0.32)	5.17 (0.57)	0.349
	HbA1C %	5.2 (0.3)	5.38 (0.3)	5.15 (0.27)	0.262
Hormones	Testosterone total (nmol/L)	1.2 (0.51)	1.57 (0.58)	1.04 (0.41)	0.051
	Prolactin	252 (126.7)	237.07 (83.1)	261.2 (146.3)	1.000
	Progesterone	5.54 (17.81)	0.41 (0.10)	8.1 (21.6)	0.001
	FSH (IU/L)	5.16 (2.23)	4.5 (1.69)	5.56 (2.47)	0.258
	LH (IU/L)	3.42 (2.45)	2.2 (1.63)	4.03 (2.58)	0.032
	AMH	24.9 (21.46)	22.05 (12.2)	26.2 (24.8)	0.883
	Free thyroxine (pmol/L)	12.81 (2.03)	13.1 (1.9)	12.71 (2.12)	0.700
	Free triiodothyronine (pmol/L)	4.15 (0.74)	3.95 (0.01)	4.28 (1.01)	0.772
	Thyroid stimulating hormone (mU/L)	7.61 (29)	2.07 (1.34)	9.75 (35.2)	0.265
Liver and kidney function	Bilirubin (μmol/L)	9.42 (5.11)	10.27 (7.29)	9.19 (4.68)	0.920
	Albumin (g/L)	46.65 (34.27)	36.14 (6.36)	51.25 (40.4)	0.058
	Alkaline phosphatase (IU/L)	67.79 (19.52)	79.85 (27.07)	62.82 (13.5)	0.085
	ALT	17.13 (13.46)	10 (3)	20.25 (15.11)	0.013
	AST	17.56 (8.83)	12.57 (2.57)	19.75 (9.75)	0.004
	Urea	3.72 (1.17)	3.72 (1.12)	3.71 (1.22)	0.792
	Creatinine	59.5 (17.02)	61 (9.59)	58.7 (19.8)	0.161
Fertility tests	Infertility duration (years)	5.02 (3.64)	3.45 (2.24)	6.1 (4.07)	0.039
	Number of follicles aspirated	15.97 (5.42)	16.76 (5.41)	15.57 (5.49)	0.500
	Number of eggs retrieved	13.36 (7.97)	14.5 (6.6)	12.76 (8.63)	0.483
	Number of eggs fertilized	7.17 (5.42)	8 (4.04)	6.76 (6.02)	0.225
	Fertility rate (%)	70.71 (23.3)	75.9 (19.93)	68 (24.81)	0.294
	Number of eggs cleaved	7.44 (5.87)	8 (4.04)	7.16 (6.69)	0.259
	Top embryo quality	4.39 (4.06)	4.92 (3.47)	4.12 (4.37)	0.258

Differences between biochemical pregnancy (yes and no) were tested by independent sample t-test (normally distributed variables) or Mann–Whitney U (variables with skewed distribution) test. Data are presented as mean (SD).

All patients underwent a standard IVF antagonist protocol (31). rFSH stimulation was started on day 2 of their menstrual cycle using Gonal-F (Merck Serono). To prevent a premature LH surge, the GnRH antagonist (Cetrotide: Merck Serono) was used. To monitor the ovarian response to stimulation, ultrasound scans were performed from day seven and every 2 days thereafter. The response to therapy was determined by follicular diameter and follicle numbers. Final maturation was initiated when two or more leading follicles were ≥18 mm using human chorionic gonadotrophin (hCG, Pregnyl, Merck Sharp and Dohme). Oocyte retrieval was performed, and the FF was centrifuged and stored at −80°C until analysis. At the same time as oocyte retrieval, an additional blood sample was taken and prepared as noted above. Transcervical embryo transfer was performed and embryos were classified using standard criteria (32): top-quality embryos on Day 3 as per Alpha Consensus (“Istanbul consensus workshop on embryo assessment: (33)proceedings of an expert meeting,” 2011). Embryo transfers were performed either on day 3 or ideally on day 5 (blastocyst) for implantation. Blood biochemistry tests were conducted at the chemistry laboratory of Hamad Medical Corporation, Doha, Qatar. Pregnancy outcomes of gestational age at delivery, birth weight, maternal weight, blood pressure, and fetal outcome were recorded. Protocols were approved by Institutional Review Boards of the Hamad Medical Corporation (15101/15) and Weill Cornell Medical College in Qatar (15-00016).

### Human Complement-Related Protein Measurements

MILLIPLEX MAP Kit Human Complement Magnetic Bead Panels 1 and 2 (HCMP1MAG-19K and HCMP2MAG-19K) were used to measure levels of 14 complement factors in the sera and FF of participants according to the manufacturer’s instructions (Merck Millipore, USA). Serum samples were diluted 200 times for complement panel 1 containing C2, C4b, C5, C5a, C9, adipsin, and mannose-binding lectin and 40,000 times for complement panel 2 containing C1q, C3, C3b/iC3b, C4, factor B, factor H, and properdin as per the manufacturer’s instructions. Five-parameter logistic regression algorithms built into the Bioplex manager six software were used to assess complement levels in reference to standards. Analysis was conducted using a Bioplex-200 instrument according to the manufacturer’s instructions (BIO-RAD, Hertfordshire, UK).

### Statistical Analysis

Comparisons were performed using *t*-test, Wilcoxon–Mann–Whitney, 1-way ANOVA, or linear models as appropriate using IBM SPSS statistics 21. Linear regression models were used when analyzing differences in complement factor levels between pregnancy outcome groups by considering age as a potential confounder. Correlations were performed using Pearson’s correlation *via* SPSS version 27. Data were presented as mean ± standard deviation (SD).

## Results

Based on their pregnancy outcome, patients were dichotomized into successful (*n* = 14) and unsuccessful (*n* = 26) pregnancies. As shown in **Table 1**, women who had successful pregnancies were slightly younger (average 4.5 years, *p* = 0.01) with higher ALT (*p* = 0.01) and AST (*p* = 0.004), but lower LH (*p* = 0.03) and lesser infertility duration (*p* = 0.04).

### Comparing Complement Factors Levels Between Successful and Unsuccessful IVF Cycles

**Figure 1:**
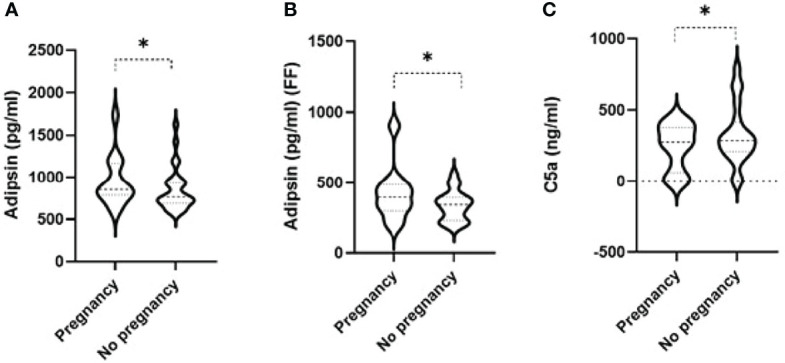
Violin plots comparing levels of adipsin in the serum prior to IVF cycle **(A)** and in follicular fluid (FF) **(B)** and of serum C5a **(C)** in women with successful and unsuccessful pregnancies. Medians and interquartile ranges are also shown within the violin plots. * signifies p-value < 0.05 from linear regression having age as a potential confounder between successful pregnancy - yes vs no.

Linear regression was used to compare levels of complement factors in the maternal serum and FF between women with successful or unsuccessful pregnancies. Among the 14 tested complement factors, levels of adipsin in the serum prior to the IVF cycle (**Figure 1A**) and FF (**Figure 1B**) were higher in women with successful pregnancies than women with unsuccessful pregnancies (*p* = 0.01 and 0.05, respectively). Conversely, serum C5a levels (**Figure 1C**) were significantly higher in participants with unsuccessful pregnancies (*p* = 0.01) compared to those with successful pregnancy (**Table 2**). When the 14 tested complement factors and levels of adipsin were measured in the serum at the time of oocyte collection, no significant changes in complement proteins were seen (data not shown).

**Table 2:** Complement factors exhibiting significant differences in sera prior to the IVF cycle and follicular fluid of women with successful compared to unsuccessful pregnancies.

Cytokine	Matrix	Estimated beta coefficient	SE	*p*-value
C5a (ng/ml)	Serum	−2.3	0.72	0.01
Adipsin (pg/ml)	Serum	0.27	0.1	0.01
Adipsin (pg/ml)	Follicular fluid	0.3	0.15	0.05

**Figure 2:**
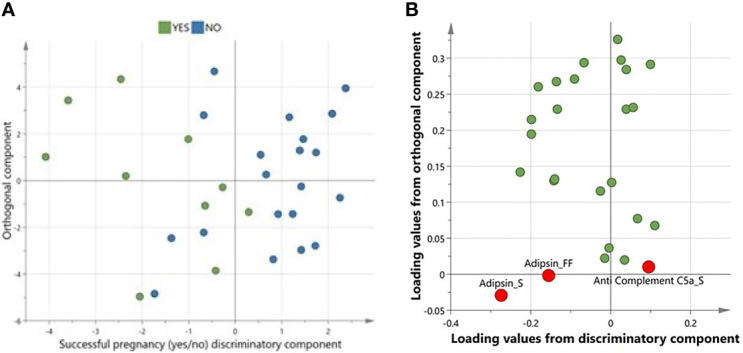
A multivariate OPLS-DA confirming complement factors differentiating successful from unsuccessful pregnancies. **(A)**Scores plot from OPLS-DA showing discriminatory and orthogonal components for successful vs unsuccessful pregnancies. **(B)** Corresponding loadings plot from OPLS-DA. Significantly different complements are highlighted in red.

A multivariate variate OPLS-DA comparing levels of complement factors in pre-IVF serum and FF between women with successful versus unsuccessful pregnancies revealed one class-discriminatory component accounting for 47% of the variation in the data due to pregnancy outcome (R-squared-Y = 0.47) (**Figure 2A**). The corresponding loading score, shown in **Figure 2B**, confirms higher adipsin levels and lower C5a levels in women with successful pregnancies compared to women with unsuccessful pregnancies.

### Multivariate Analysis of Pre-IVF Serum and FF Comparison With Pregnancy Outcome

A multivariate variate OPLS-DA comparing levels of complement factors in pre-IVF serum and FF between women with successful versus unsuccessful pregnancies revealed one class-discriminatory component accounting for 47% of the variation in the data due to pregnancy outcome (*R*-squared-*Y* = 0.47) (**Figure 2A**). The corresponding loading score, shown in **Figure 2B** confirms higher adipsin levels and lower C5a levels in women with successful pregnancies compared to women with unsuccessful pregnancies.

### Correlation of Adipsin and C5a With Covariates

**Figure 3:**
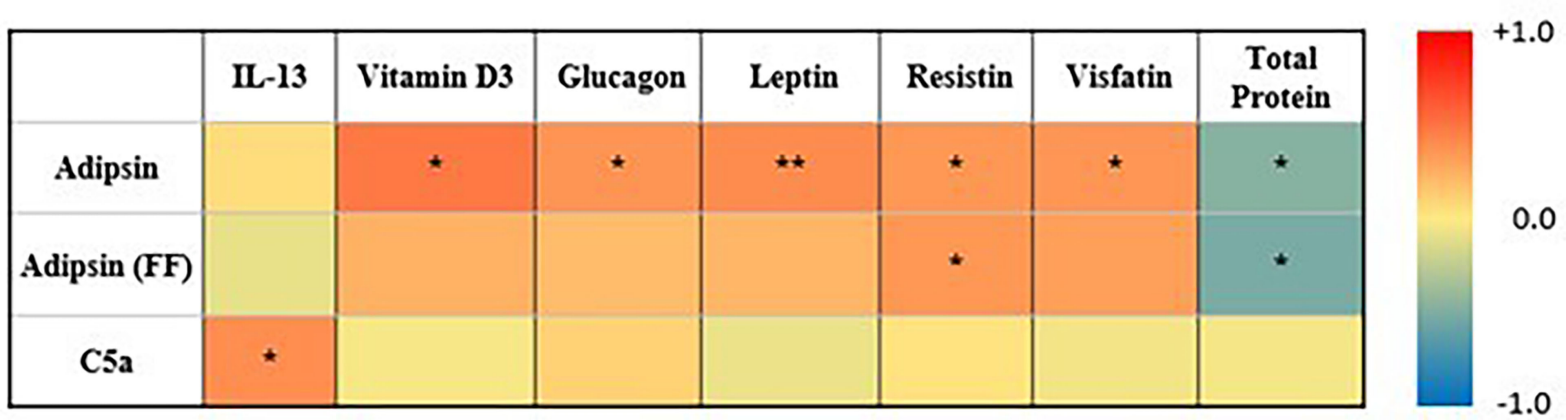
Heatmap summarizing correlation between adipsin (serum and FF), C5a (serum and FF), and inflammatory markers. Colors reflect the range of correlation coefficient (−1.0 in blue to +1.0 in red). * signifies p-value < 0.05, ** signifies p-value < 0.01.

Pearson’s correlation between levels of adipsin (serum and FF), C5a, and various inflammatory and metabolic disease markers showed positive correlations between pre-IVF serum adipsin and levels of vitamin D (*R* = 0.5, *p* = 0.02), glucagon (*R* = 0.4, *p* = 0.03), leptin (*R* = 0.4, *p* = 0.01), resistin (*R* = 0.4, *p* = 0.02), and visfatin (*R* = 0.4, *p* = 0.02), but a negative correlation with total protein (*R* = −0.5, *p* = 0.03). Similarly, adipsin levels in FF also showed positive correlation with resistin (*R* = 0.4, *p* = 0.05) and a negative correlation with total protein (*R* = −0.5, *p* = 0.03). C5a was only significantly correlated with IL-13 (*R* = 0.4, *p* = 0.04) (**Figure 3**).

### Comparing Complement Factors Levels Associated With the Number of Top-Quality Embryos

**Figure 4:**
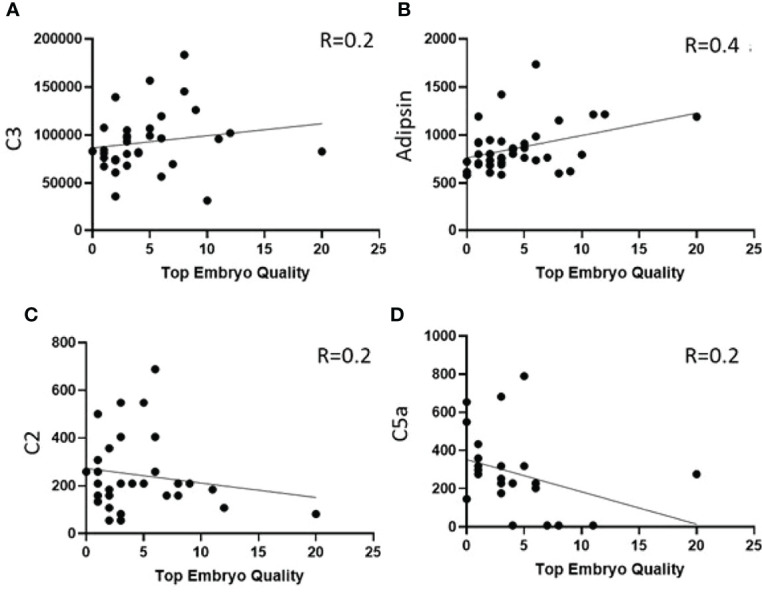
Correlation between number of top-quality embryos and complement factors in pre-IVF serum. **(A, B)** Showing increased levels of C3 and Adipsin with high numbers of top embryo quality and **(C, D)** shows decreased levels of C2 and C5a with high numbers of top embryo quality.

Linear regression was used to identify the association between complement factors in the pre-IVF serum and FF and the number of top-quality embryos (ordinal). Higher numbers of top-quality embryo levels were associated with increased levels of C3 (**Figure 4A**), properdin, C1q, factors H and B, C4, and adipsin (**Figure 4B** and **Table 3**). Conversely, C2 levels were reduced with higher numbers of top-quality embryos (**Figure 4C**). Interestingly, C5a was also negatively correlated with the number of top-quality embryos (*R* = 0.2, *p* = 0.05) (**Figure 4D**).

**Table 3:** Complement factors exhibiting significant differences in pre-IVF sera of women generating the highest number of top-quality embryos.

Cytokine	Matrix	Estimated beta coefficient	SE	*p*-value
C-3 (ng/ml)	Serum	2.1	3.8	0.0018
Properdin (ng/ml)	Serum	1.8	3.5	0.0037
C1q (ng/ml)	Serum	1.9	3.5	0.0038
Factor H (ng/ml)	Serum	1.8	3.3	0.0048
Factor B (ng/ml)	Serum	1.7	3.2	0.0059
C2 (ng/ml)	Serum	−1.0	−3.0	0.0061
C4 (ng/ml)	Serum	1.8	3.1	0.0071
Adipsin (pg/ml) A	Serum	0.2	2.9	0.0081

### Comparison of Complement Factors in Serum and FF With Embryo Quality

**Figure 5:**
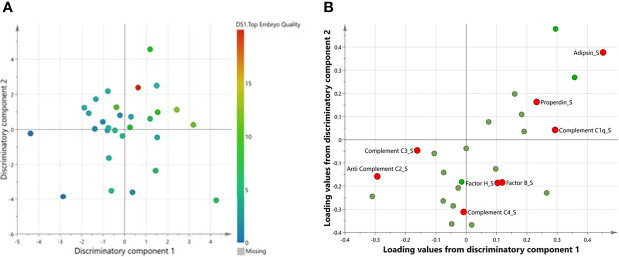
A multivariate OPLS confirming complement factors associated with number of top-quality embryos. **(A)** Scores plot from OPLS-DA showing two discriminatory components for top embryo quality. **(B)** Corresponding loadings plot from OPLS-DA. Significantly different complements are highlighted in red.

A multivariate OPLS analysis comparing levels of complement factors in serum and FF between women with number of top-quality embryos revealed one class-discriminatory component accounting for 44% of the variation in the data due to pregnancy outcome (*R*-squared-*Y* = 0.44) (**Figure 5A**). The corresponding loading score, shown in **Figure 5B**, confirms greater levels of serum C3, properdin, C1q, factors H and B, C4, and adipsin, but lower C2, with higher numbers of top-quality embryos (**Figure 4C**).

## Discussion

It is critical to identify novel biomarkers that may identify those women who will have a favorable outcome and those who will not from those who are undergoing assisted reproductive technology; this may guide the therapeutic strategy. Whereas serum may offer a general indication of the woman’s health, the FF during IVF treatment reflects the intimate environment of the developing cumulus/oocyte complex (34). Pregnancy activates the complement system by triggering the innate immune response, leading to elevation in the levels of C3a, C4a, and C5a. This elevation compensates for the inhibition of adaptive immunity during the normal pregnancy (24) and plays a critical role in pregnancy outcome (35). However, reports have indicated that circulating levels of C3a and C5a pose increased risk of preeclampsia (36). The interface between maternal and fetal tissues is enriched with complement inhibitors to protect the placenta from consequences of complement activation and adverse pregnancy outcomes (37). Complement components are also present in the mucosal secretions of the fallopian tubes, cervix, and uterus (38), suggesting that pregnancy failure could potentially result from excessive complement activation in the pre-implantation stage (35). In this study, we have assessed the levels of complement factors in the pre-IVF serum, serum at the time of oocyte retrieval, and FF samples obtained from women undergoing IVF cycles and compared their levels in relation to IVF outcome (i.e., successful or unsuccessful pregnancy and number of top-quality embryos). Out of 14 assessed complement factors, only adipsin and C5a were significantly associated with IVF outcome. Higher levels of pre-IVF serum and FF adipsin and lower levels of serum C5a were identified in successful pregnancies. In order to understand the potential mechanisms underlying these associations, levels of various cytokines, adipokines, and myokines were determined in the pre-IVF serum and FF samples from the same women and compared in relation to IVF outcome. Serum adipsin levels were positively correlated with circulating levels of vitamin D, glucagon, leptin, resistin, and visfatin, but negatively correlated with total protein. Higher numbers of top-quality embryos were associated with increased levels of C3, properdin, C1q, factors H and B, C4, and adipsin, but with reduced C2 and C5a levels. However, serum taken at the time of FF removal showed no correlation of the complement proteins or with any other parameters, suggesting that the intervention of the exogenous gonadotrophin therapy and the hormonal changes that resulted in oocyte stimulation have modified the complement system abrogating the predictive indices seen in the pre-IVF sera for these patients. The effect of exogenous gonadotrophin therapy on the complement system during oocyte stimulation is not known and needs further specific investigation.

The higher levels of adipsin in the pre-IVF sera and FFs taken from women with successful pregnancies suggest a positive role of adipsin in the pregnancy outcome. The higher serum levels of adipsin prior to implantation could reflect a protective phenotype since serum adipsin levels are negatively associated with insulin resistance, especially in overweight and obese subjects (39). Studies have indicated that adipsin improves the maintenance of β-cell function as adipsin knockout mice exhibit glucose intolerance due to insulinopenia while replenishment of adipsin boosts their insulin secretion (9). Indeed, studies have shown that insulin resistance lowers implantation rate in the *in vitro* maturation/IVF embryo transfer cycle (40). Therefore, lower adipsin in failed cycles could reflect insulin resistance-mediated lowering of the maturation/IVF embryo transfer cycle. Our data showed that serum adipsin levels were positively correlated with serum vitamin D, glucagon, and various adipokines such as leptin, resistin, and visfatin. However, other studies suggested a negative role for adipsin in pregnancy being associated with metabolic changes during pregnancy (41, 42), including pathogenesis of preeclampsia due to its role in activation of factor B with a direct association with the development of preeclampsia (43, 44).

Several studies have indicated the important physiological role of adipocytokines in metabolism (45). Leptin was suggested to be involved in the control of reproductive functions by acting both directly on the ovaries and indirectly on the central nervous system (46), although other reports have suggested no correlation with pregnancy outcome (47). Reports have also shown that resistin does not correlate with IVF outcome (48). Visfatin, on the other hand, was found to restore ovarian aging and fertility in aging mice (49). Furthermore, previous studies have shown that women with elevated vitamin D had more successful IVF cycles (50). Inflammation is another critical factor for embryo implantation during pregnancy. A proinflammatory environment is essential during embryo implantation (51), followed by suppression of inflammation for the rest of the pregnancy until onset of labor. However, if chronic or acute inflammation persists for a longer duration within the uterine cavity, this may increase the chance of spontaneous abortion or preterm labor. Obese women are likely to have a low-grade inflammatory state throughout pregnancy, which may compromise embryo implantation. This may explain why the success rate of IVF-assisted pregnancy is lower in obese women.

Our data showing higher levels of C5a in the sera taken from women with unsuccessful pregnancies suggest a negative role for C5a in the pregnancy outcome. Our data therefore are in agreement with previous studies implicating elevated C5a levels in trophoblast dysfunction, impaired placental angiogenesis, and adverse pregnancy outcomes (36, 52, 53). Higher levels of C5a were also associated with preterm birth by increasing contraction frequencies (35), cervical remodeling, and fetal brain injury (54–56). Therefore, higher maternal C5a serum levels could be an indicator of failed IVF cycles, as suggested by our data.

Our data also indicate that higher numbers of top-quality embryos are associated with increased levels of C3, properdin, C1q, factors H and B, C4, and adipsin, but with reduced C2 and C5a levels. Levels of specific substances in FF were previously shown to be associated with fertilization outcome and early post-fertilization development, including elevated levels of LH, growth hormone (GH), prolactin, 17β-estradiol (E2), and insulin-like growth factor (IGF)-I and lower IL-1 in women with successful pregnancies. Furthermore, LH and GH levels were higher in follicles, resulting in top-quality embryos with the best morphology and fastest cleavage rate (57). The immunomodulatory role of locally produced complement factors, including C3, properdin, C1q, C4, adipsin, and factors H and B, in immunological tolerance and cellular survival was previously established (58–60). The positive correlation between top-quality embryos and these complement factors could reflect a pro-survival environment for developing oocytes that resulted in better-quality embryos. Future studies are warranted to investigate the functional relevance of these associations in larger independent cohorts.

A strength of this study was that our cohort does not include known causes of infertility, but further studies on specific causes of infertility are needed. The fact that the study utilized pre-IVF and matched serum at the time of oocyte retrieval when the FF samples were taken is also a major strength. Limitations include the fact that our complement cascade panel only had 14 complement-related protein markers and so other crucial proteins involved in the activation of complement cascades were not measured, including protein D hydrolysis and activation mechanisms associated with the MASP proteases. Our pilot study was undertaken in a small cohort of women, and larger studies are needed in the future to confirm and extend our findings. In addition, all the women were Qatari and, therefore, the results may not be generalizable to other ethnic groups.

## Conclusions

This study reports, for the first time, higher adipsin and lower C5a levels in the pre-IVF serum in successful IVF-assisted pregnancies and showed positive correlations between complement factors and embryo quality, with a potential utilization as predictive biomarkers of successful pregnancies in obese women.

## Data Availability Statement

The original contributions presented in the study are included in the article/**Supplementary Material**. Further inquiries can be directed to the corresponding author.

## Ethics Statement

The study was approved by the Institutional Review Boards of the Hamad Medical Corporation (15101/15) and Weill Cornell Medical College in Qatar (15-00016) research Ethics Committee, and all study participants signed an informed consent form prior to participation. The patients/participants provided their written informed consent to participate in this study.

## Author Contributions

MR designed the study and performed the measurements. ID and NR analyzed the data and performed statistical analysis and prepared the tables. YD, LA, TA, MS, MB, EE, HB, and SA supervised clinical studies, recruited the patients, and collected demographics. AB prepared pathway analysis for the manuscript. SA, MR, AA-S, and ME supervised the study and interpreted data. ME wrote the manuscript. All authors contributed to the article and approved the submitted version.

## Conflict of Interest

Authors MR, TA, MS, MA, HB, and AA-S were employed by Hamad Medical Corporation.

The authors declare that the research was conducted in the absence of any commercial or financial relationships that could be construed as a potential conflict of interest.

## Publisher’s Note

All claims expressed in this article are solely those of the authors and do not necessarily represent those of their affiliated organizations, or those of the publisher, the editors and the reviewers. Any product that may be evaluated in this article, or claim that may be made by its manufacturer, is not guaranteed or endorsed by the publisher.
