# Supplementary figures and images for: Elevated Adipsin and Reduced C5a Levels in the Maternal Serum and Follicular Fluid During Implantation Are Associated With Successful Pregnancy in Obese Women
Source: Front Endocrinol (Lausanne). 2022 Jul 13;13:918320. doi: 10.3389/fendo.2022.918320 (PMC9326155; doi:10.3389/fendo.2022.918320)

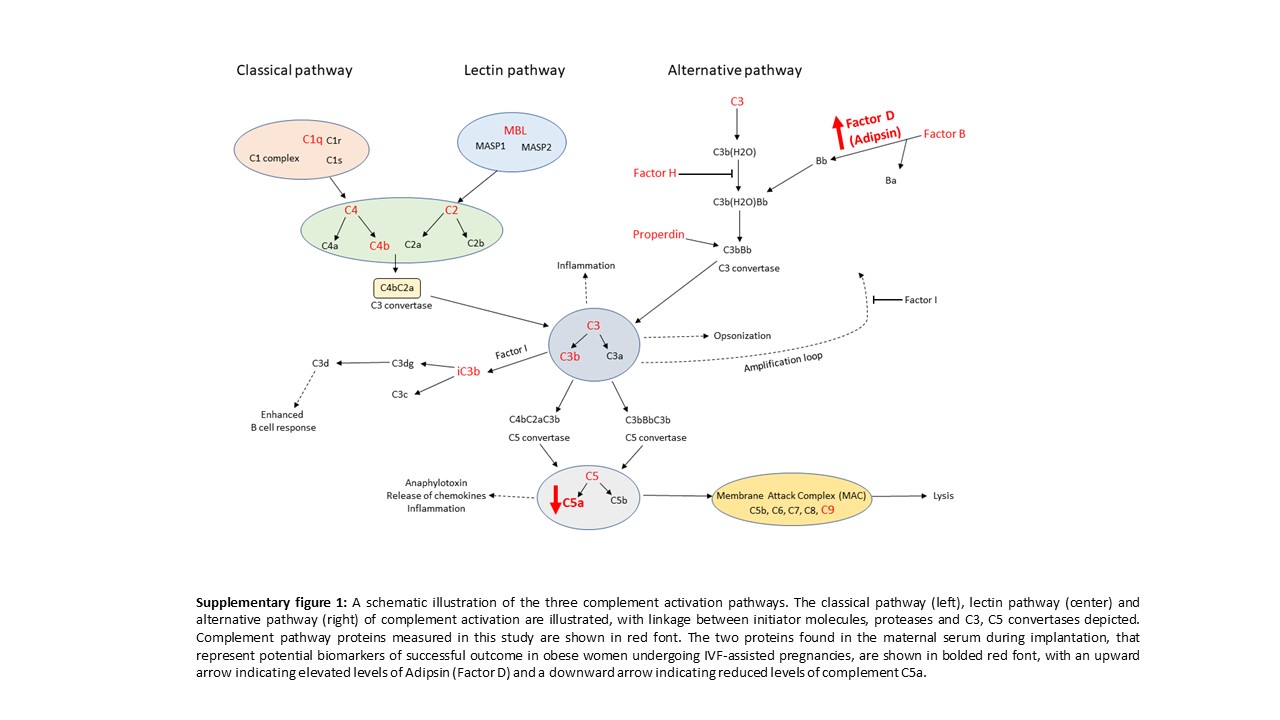

Supplement: Supplementary file 1 [file Image_1.jpeg]
